# Impact of maximal physical exertion on interference control and electrocortical activity in well-trained persons

**DOI:** 10.1007/s00421-018-3977-x

**Published:** 2018-08-30

**Authors:** Thomas Finkenzeller, Michael Doppelmayr, Sabine Würth, Günter Amesberger

**Affiliations:** 1 Paris Lodron University of Salzburg, Hallein, Salzburg Austria; 2 Johannes Gutenberg University of Mainz, Mainz, Germany

**Keywords:** Graded exercise test, Physical exhaustion, Executive function, Event-related potentials, Recovery period

## Abstract

**Purpose:**

The aim of this study was to examine the impact of a maximal physical load on cognitive control in twelve well-trained males focusing on the time course of changes in a 15 min post-exercise interval.

**Methods:**

Prior to and three times after an incremental cycle ergometer task until exhaustion, behavioural performance and neurophysiological correlates using N2 and P3 event-related potentials (ERPs) were assessed during the execution of a modified flanker task. These data were compared to a control condition following the same protocol, however, without physical load between pre-test and post-tests.

**Results:**

Regardless of compatibility (congruent, incongruent), behavioural findings revealed a significant interaction of Condition × Time with shorter reaction times in the post-exercise blocks as compared to the control condition. Neuroelectric measures demonstrated exercise induced effects of a reduced central N2 amplitude and shorter parietal P3 latency in the time course of post-exercise flanker blocks as compared to rest.

**Conclusions:**

It is concluded that a state of maximal physical exhaustion facilitates information processing speed in a cognitive control task in well-trained persons. This effect persists even after a recovery period of 15 min. The current findings contribute to a deeper understanding of the neuronal mechanisms of interference control following maximal physical load, suggesting a reduced conflict monitoring as indicated by a reduced N2 amplitude and an increased stimulus classification speed as reflected by P3 latency. The flanker task, however, might have been too simple to elicit monitoring conflicts on the behavioural level.

## Introduction

Maintaining cognitive control in a state of physical exhaustion is essential to be successful in many competitive sports. According to Tillman and Wiens (2011) cognitive control refers to “the ability to select those aspects of a situation that are relevant to one’s current goal and let actions be guided by this information, while ignoring other, goal-irrelevant aspects” (p. 1405). Keeping a high level of cognitive control is not only important during exercise, but also in phases of low physical load. For example, a biathlete interrupts cross-country skiing with no recovery phase between running and shooting. While aiming, the athlete attempts to fade out irrelevant factors (e.g. opponents, background noise…), and has to be aware of shooting performance relevant factors (e.g. changing weather conditions, shooting pattern, rifle set up...). As another example, team sport athletes obtain feedback and tactical instructions in time out phases or in the halftime. In these periods, game relevant information is provided, and game irrelevant information must be ignored (e.g. announcements, insults from spectators…). All of these inhibitory cognitive processes occur under the impact of physical arousal caused by the foregoing physical load. Thus, as an example, there is still uncertainty whether it is best to coach at the beginning, in the middle or at the end of a 15 min halftime regarding inhibition of irrelevant information and speed of information processing.

The flanker task (Eriksen and Eriksen 1974) is commonly used in research on the relationship between exercise and cognitive control. In a flanker task, the reaction time to a central target (the midposition item e.g. >) that is flanked either by items identical to the target (e.g. >>>>>; congruent condition) or by the same stimuli but in the opposite direction (e.g., >><>>; incongruent condition) is assessed. In incongruent conditions, the reaction time is generally increased and reactions are less accurate (Eriksen and Schultz 1979). The difference in reaction time between incongruent and congruent trials, which is termed flanker effect (Purmann et al. 2011), reflects the required time to solve the conflict evoked by the incongruent flanking stimuli (Tillman and Wiens 2011).

Few studies investigated cognitive performance immediately following maximal physical exhaustion using graded incremental cycling or treadmill running tests. In previous studies, an inconsistent pattern emerged, suggesting a selective impact of maximal physical exhaustion on a subsequent cognitive performance (Covassin et al. 2007; Thomson et al. 2009; Llorens et al. 2015; Coco et al. 2009). To our knowledge, cognitive control was only addressed after maximal physical exhaustion by Kamijo et al. (2004b) using a go/no-go task, which requires to react as fast as possible on a target signal and to inhibit motor reaction to a non-target signal. Pre-motor time was unaffected by a preceding physical exercise load regardless of intensity. Neuroelectric measures in terms of the P3, however, showed a decrease of amplitude after maximal physical exhaustion, an increase after moderate exercise intensity, and no change after low intensity in comparison to a non-exercise control condition, leading to the suggestion that P3 amplitude changes in an inverted U-shaped behaviour as a function of exercise intensity.

The P3 component is a positive deflection in the stimulus-locked event-related brain potential (ERP) of the human electroencephalogram (EEG) that occurs at central and parietal sites 250–500 ms after stimulus onset. P3 amplitude is used to index the allocation of cognitive resources during stimulus engagement, and P3 latency is thought to reflect stimulus classification speed—the time required to detect and to evaluate a stimulus (Polich 2007; Donchin and Coles 1988). Thus, this component is a promising index (Hillman et al. 2003; Olson et al. 2016), which may contribute to a deeper understanding of the interplay between exercise and inhibitory processes. Another important EEG parameter, the N2 is characterized as a negative peak at frontocentral sites around 200–350 ms after stimulus onset and is interpreted as an index of conflict monitoring (Purmann et al. 2011), resolving a reaction conflict (Kopp et al. 1996), and mismatch between a stimulus and a template (Folstein and Van Petten 2008).

Several ERP studies have used the flanker task to examine the effects of a preceding low or moderate physical exercise bout on cognitive control (Chang et al. 2015; Hillman et al. 2003; Kamijo et al. 2007, 2009; O’Leary et al. 2011; Themanson and Hillman 2006). Kamijo et al. (2007) reported that P3 amplitude of an EF task increased immediately after light and moderate cycling, and decreased after hard exercise to baseline level, supporting the hypothesis of an inverted U-relationship between exercise intensity and P3 amplitude. In discrepancy to arousal theories (for overview see Lambourne and Tomporowski 2010), P3 latencies of incongruent trials and pre-motor reaction time were significantly shorter in comparison to baseline—regardless of the preceding exercise intensity.

Despite numerous studies on the relationship between exercise and cognition, there is only limited knowledge on cognitive performance following graded incremental exercise until maximal exhaustion, in particular on interference control—an executive subcomponent “geared towards managing the disruptive effects of irrelevant information on the active maintenance of task goals” (Burgess et al. 2011). Thus, interference control appears to have a significant role in sports because it has to be maintained even under highest cardiovascular load. It is still unclear in which way maximal exhaustion affects interference control, and how interference control changes in the time course of recovery. To address this question, the time course of changes in cognitive control was compared between a non-exercise and an exercise condition by applying a 2 (condition) × 4 (measurement) within-subject research design. Well-trained persons performed a flanker task prior to (*t*1) and three times (*t*2–*t*4) after a graded incremental cycling test until maximal exhaustion and after a break of 17 min without physical load in-between. Recovery times of no (*t*2), short (*t*3; 3.25 min), and middle duration (*t*4; 11.5 min) were considered with the aim to assess the impact of the duration of a recovery period on behavioural and neuroelectric measures.

Referring to Kamijo et al. (2007), reaction time on congruent and incongruent items was assumed to decrease in all three EF blocks of the recovery phase (*t*2–*t*4) as compared to pre-exercise (*t*1). According to arousal theories (Davey 1973), it was expected that changes of physiological arousal within the recovery phase would go along with alterations in reaction times among the three post-exercise measurements. The simplicity of the applied Eriksen flanker (EF) task causing a ceiling effect (McMorris and Hale 2012) would result in no alterations of error rate in the post-exercise EF blocks (*t*2–*t*4). The flanker effect was hypothesized to benefit from high physiological arousal (O’Leary et al. 2011) as a result of a more pronounced reduction of reaction time on incongruent as compared to congruent items (Chodzko-Zajko 1991). With an increase of recovery time, the impact of the more reduced reaction time on incongruent items might disappear leading to an increased flanker effect score.

According to the assumption of an inverted U-shaped relationship between exercise intensity and P3 amplitude (Kamijo et al. 2007), P3 amplitude was expected to be very similar during baseline condition (t1) (Kamijo et al. 2004a) and immediately following a maximal possible workload (*t*2). The short (*t*3) and medium recovery time (*t*4) would lead to an increase P3 amplitude elicited by a decline of physiological arousal.

P3 latency reflecting stimulus classification speed was considered shorter after maximal physical exhaustion (*t*2) as compared to baseline (*t*1). According to Kamijo et al. (2007), it was expected that the decrease in P3 latency will be more pronounced on incongruent stimuli. Regarding the EF blocks with a short (*t*3) and medium recovery period (*t*4), P3 latency was assumed different from *t*2 due to a decrease of physiological arousal.

Using an exploratory approach, the N2 component was assessed for the first time after maximal physical exhaustion with the aim to get insights into processes of conflict monitoring.

## Methods

### Participants

Sample size was a priori determined with the computer program Gpower 3.1.9.2 (Faul et al. 2007). A power analysis indicated that a total sample of 12 people would be needed to detect medium effects (*f* = 0.25) with 80% power and alpha of 5% considering a 2 (conditions) × 4 (measurements) ANOVA with repeated measures. In this analysis, correlation amongst repeated measures was set at 0.8, which was obtained in a pilot study on test–retest reliability (*n* = 56; short-term interval of 1 min; see Table 2) of the applied EF task. Thus, a sample of 12 well-trained, right-handed males was recruited engaging in various sports (mountain biking, mountaineering, triathlon, soccer, running, tennis, ice hockey, etc.). The participants had to exercise regularly three times or more per week for a total exercise time of at least 4 h per week. Females were not considered to control for hormonal changes (Becker et al. 1982) and different emotional ways of coping with a physical stressor (Crocker and Graham 1995). Table 1 shows age, anthropometrical, physiological and training characteristics of the participants. At the beginning, the subjects signed an informed consent. The participants confirmed that they were healthy and did not suffer from any neurological disorders or cardiovascular diseases. Furthermore, they were instructed to take care to sleep sufficiently, to drink sufficiently prior to the testing, and to avoid hard training sessions the day before the measurements as well as to abstain from caffeine 2 h before the measurements. The local ethics committee approved the study protocol.

**Table 1:** Anthropometrical, physiological and training characteristics of participants

	*M*	SD
Age (years)	27.66	7.39
Height (cm)	177.25	6.68
Weight (kg)	75.33	7.20
*W*_max_/kg	4.49	0.66
HR_max_	183.83	13.04
RPE at maximal exhaustion	18.58	1.31
Training session per week	4.08	1.51
Hours of training per week	7	3.02
Training on bike per week (h)	1.83	3.33

### Procedure

**Fig. 1:**
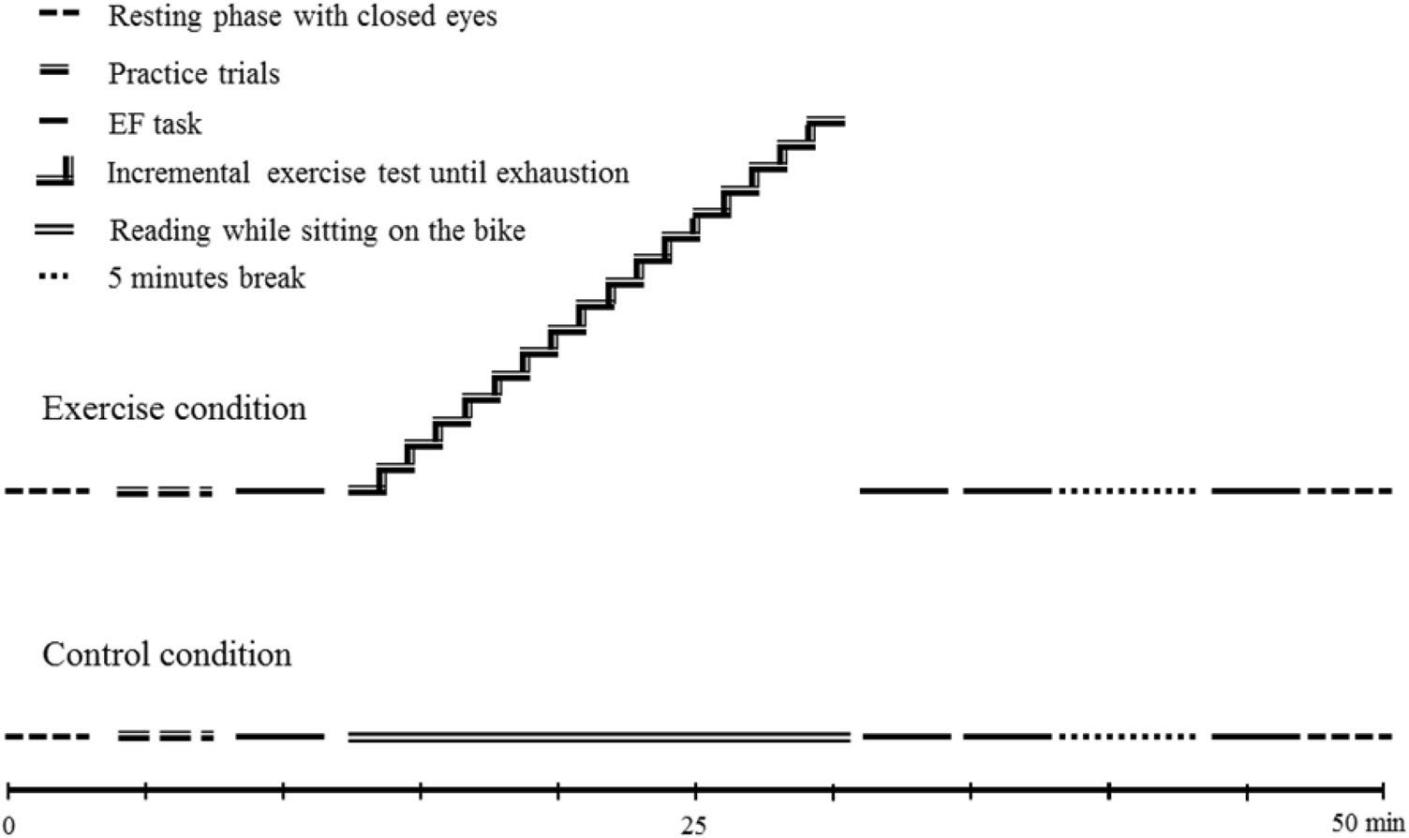
Representation of the experimental sessions that consisted of performing an EF task prior to and three times after an incremental exercise task until exhaustion (of 17 min in this figure) and a control condition without physical load, respectively

Each participant visited the laboratory three times in a period of 7 to 14 days. On the first visit, the participants were familiarized with the EF task by performing twenty practice trials after a brief introduction. Subsequently, three EF blocks were administered. The compilation of the EF block is described in detail in the measure section. Within 4 days after familiarization, an exercise condition or a control condition was conducted in counterbalanced order within 5 to maximal 13 days (*M* = 7.33, SD = 1.93 days). In both conditions, the procedure was identical, and the daytime of testing was kept constant to control for circadian rhythmicity effects. The experiment started with a resting phase of 3 min, in which the participants sat on the cycle ergometer and closed their eyes. Subsequently, an EF practice block of 120 trials was conducted followed by one EF block (see section “EF task”; *t*1) prior to the experimental condition (cycling or reading). Immediately after the cessation of the experimental conditions, participants performed two EF blocks (*t*2 and *t*3), and a final EF block after a break of five minutes (*t*4) to obtain knowledge on the impact of no (*t*2), short (*t*3) or medium (*t*4) recovery time on interference control. The procedure ended with a resting phase that was identical to the resting phase at the beginning. Figure 1 gives a schematic representation of the procedure.

### Incremental maximum exercise condition

The incremental exercise test was performed on an ergoselect 100 bicycle ergometer (comp. ergoline, Germany) equipped with an eddy current brake ensuring that work rate is independent of revolution speed. The test started at a physical load of 40 Watts (W) with an increment of 20 W/min until exhaustion (Wonisch et al. 2008). This lasted between 11 and 25 min (*M* = 16.42, SD = 3.34) regarding the fitness level of the subject. During the exercise test, qualified persons continuously monitored well-being and physiological parameters of the participant. The participants cycled at their individually preferred cadence that was controlled for stability during the whole exercise test by the experimenter. The subjects could also monitor the cadence on a screen that was fixed towards the handlebars. All participants selected between 70 and 90 revolutions per minute. There was no situation to report critical changes in cadence to the participants. The aim of the incremental exercise task was to maintain the individual preferred cadence for as long as possible. The test was terminated when the participant could no longer sustain the required power output at their individual preferred cadence whilst staying seated in the saddle (Robertson and Marino 2015).

### Control condition

The control condition lasted 17 min to provide a comparable time interval as required to perform the exercise test. The duration of the incremental exercise test was estimated based on data of the pilot study. During this period, the participants sat on the ergometer without pedalling and had to read in a bicycling magazine.

### Measures

#### Rate of perceived exertion (RPE)

The RPE was assessed with the Borg scale (Borg 1998) immediately after break-off of cycling to control for maximal physical exhaustion. The scale ranges from 6 (no exertion at all) to 20 (maximal exertion).

#### EF task

A modified version (Hillman et al. 2006, 2009; Pontifex and Hillman 2007) of the EF task (Eriksen and Eriksen 1974) was applied in this study. Short-term test–retest reliability was determined in advance with a sample of 56 male and female sport students. Findings on reliability are provided in Table 2. Aim of the task is to indicate the direction of an arrow by reacting as fast and as precise as possible. An external numeric keypad (ednet model no: SKP-20) was used to give the respective reaction with the left and right thumbs (left arrow = numeric key for 4; right arrow = numeric key for +) that were placed throughout the whole EF block on the corresponding keys. In the congruent condition, the target arrows were flanked by two identical arrows on the right and on the left showing in the same direction (>>>>> or <<<<<). In the incongruent condition, the flanking stimuli were presented in the opposite direction of the target arrows (<<><<or >><>>). The arrows were displayed in white colour on a black background (arial 70 points font size; width: 13 mm, height: 12 mm). A block consisted of 50 congruent and 50 incongruent trials that were administered in randomized order. The stimuli were presented for 150 ms, and the inter-stimulus interval was randomized with periods of 1500, 1750, or 2000 ms to avoid anticipatory strategies. Responses were recorded during the whole period of a trial. An EF block lasted approximately 3 min and 15 s. The monitor was positioned 60 cm in front of the head. The software presentation (Neurobehavioral Systems, Albany, USA) was used to present the stimuli and to set markers in the EEG recording.

**Table 2:** Intraclass correlation coefficient of reliability (ICC; 2-way mixed model), 95% confidence interval (CI) of ICC, and standard error of measurement (SEM)

*n* = 56	ICC	95% CI	SEM (ms)
Reaction time of correct reactions on congruent items	0.86	0.75–0.92	13.95
Reaction time of correct reactions on incongruent items	0.85	0.75–0.91	18.44
Number of errors on incongruent items	0.89	0.81–0.93	1.45
Flanker effect	0.73	0.57–0.83	14.50

#### Data recording, pre-processing and data reduction

A predefined 21-channel EEG, a bipolar electrooculogram (EOG; vertical and horizontal) and a bipolar electrocardiogram (ECG) were recorded and digitized at 512 Hz (24-bit solution) using electrodes with carbon coating and active shielding (NeXus-32, Mind Media B. V., Herten, Netherlands). The ground electrode was placed at AFz and a linked ear mastoid reference was applied.

The psychophysiological data were analysed using the software Brain Analyzer 2.1 (Brain Products, Gilching, Germany). The EEG signals were band-pass filtered (IIR filter) from 0.5 to 50 Hz (48 dB/octave), notch filtered (50 Hz), and then subjected to an ocular correction using independent component analysis (Infomax algorithm) based on the horizontal and vertical EOG. EEG data were screened automatically and then visually for artifacts. The criteria for the amplitude range was set between ± 100 µV and the maximal allowed voltage step was defined by 50 µV/ms. Stimulus-locked epochs from − 100 to 1000 ms around the stimuli were created. Baseline correction for ERPs was performed using the 100 ms pre-stimulus period (Pontifex and Hillman 2007; Kamijo et al. 2007; Olson et al. 2016). N2 and P3 components were determined automatically and subsequently visually controlled. Inspection of the single-subject average waveforms indicated that the N2 component peaked between 150 and 250 ms poststimulus, and the P3 component 250–350 ms poststimulus for each subject. Therefore, the N2 amplitude was defined as the maximal negative deflection of the baseline adjusted interval of 150 and 250 ms after stimulus onset. P3 amplitude was quantified as the maximum positive deflections occurring within 250–350 ms after stimulus onset. N2 and P3 latency were defined as the time from the stimulus onset to the peak of the N2 and P3 amplitude, respectively. Visual inspection of individual ERPs indicated that N2 amplitude was most pronounced at sites C3, Cz, and C4, and P3 amplitude was most visible at P3, Pz, and P4. Thus, the amplitudes and latencies of these highly correlated positions were averaged to get more reliable components in accordance to Tillman and Wiens (2011). Hence, mean amplitude and mean latency of correct trials was used as dependent measure of N2 (average of C3, Cz, and C4), and P3 (average of P3, Pz, and P4). Incorrect trials were not analysed because of a too low number of cases. ECG data were analysed using the software Kubios HRV 2.2 (Biosignal Analysis and Medical Imaging Group, Kuopio, Finland). The mean average heart rate (HR) for each EF block was computed for a time interval of 3 min ending with the finalisation of the task. The mean average HR of the last 30 s prior to the break-off of the cycling was evaluated for the assessment of heart rate at physical exertion (HR_max_).

### Data analysis

For each EF block, the mean reaction times of correct reactions on congruent and incongruent reactions as wells as the number of errors on incongruent items were analysed. Furthermore, the difference in the mean reaction time of correct reactions between incongruent and congruent stimuli (flanker effect) was computed to obtain a comprehensive picture of interference control.

Three-way analyses of variance (ANOVAs) with repeated measures were used to analyse changes in reaction time as well as alterations in amplitude and latency of N2 and P3, in which Condition [control vs. exercise], Time [EF blocks at *t*1–*t*4] and Compatibility [congruent vs. incongruent] were within subject factors. Whenever an interaction of Condition × Time was obtained, subsequent 4 × 2 (Time [*t*1–*t*4] × Compatibility [congruent, incongruent]) ANOVAs were computed for each condition separately. In case of a significant result for Time, post hoc analyses were conducted using Bonferroni adjustment.

For the number of errors on incongruent items and on the flanker effect, 4 × 2 (Time [*t*1–*t*4] × Condition [control vs. exercise]) ANOVAs were run. In case of a significant interaction of Time × Condition, an univariate ANOVA with the factor Time was computed for each condition separately. Significant Time effects were subsequently analysed by Bonferroni adjusted post hoc tests.

Regarding all ANOVAs, Greenhouse-Geisser *p*-values are reported, if a violation of sphericity was observed. Alpha level for significance was set at 0.05. Effect sizes were calculated using partial Eta square (*η*_p_^2^). All analyses were conducted using the software IBM SPSS Statistics for Windows (Version 23.0; IBM Corp., Armonk, NY, USA).

## Results

Table 3 shows means (*M*) and standard deviations (SD) of heart rate, and parameters of the EF task for pre-exercise (*t*1) and the three post-exercise EF blocks (*t*2–*t*4).

**Table 3:** Heart rate and EF performance data of the pre- (*t*1) and post-intervention EF blocks (*t*2–*t*4)

	Control condition		Exercise condition	
	*M*	SD	*M*	*SD*
Heart rate (beats/min)				
BL	62.15	6.52	65.15	6.52
*t*1	69.93	12.90	67.46	10.56
*t*2	65.81	10.76	125.86	16.81
*t*3	65.68	9.16	104.89	13.87
*t*4	63.98	7.49	98.62	10.55
Reaction time of correct reactions on congruent items (ms)				
*t*1	364.19	30.02	363.19	24.30
*t*2	363.88	25.03	349.06	24.75
*t*3	361.36	26.22	352.15	21.52
*t*4	366.37	22.01	351.11	23.75
Reaction time of correct reactions on incongruent items (ms)				
*t*1	438.63	37.42	432.97	30.57
*t*2	438.55	38.47	416.37	27.95
*t*3	433.64	37.69	412.12	34.55
*t*4	429.98	35.22	407.56	31.65
Number of errors on incongruent items				
*t*1	4.33	3.17	4.33	3.96
*t*2	3.08	2.19	4.83	2.89
*t*3	2.42	2.15	3.83	2.86
*t*4	4.08	2.57	3.67	2.35
Flanker effect (ms)				
*t*1	74.44	28.61	69.78	22.79
*t*2	74.67	29.87	67.31	19.81
*t*3	72.28	21.27	59.96	19.61
*t*4	63.60	24.13	56.46	16.58

Baseline (BL) = 3 min resting phase at the beginning

### Behavioural performance on EF task

**Fig. 2:**
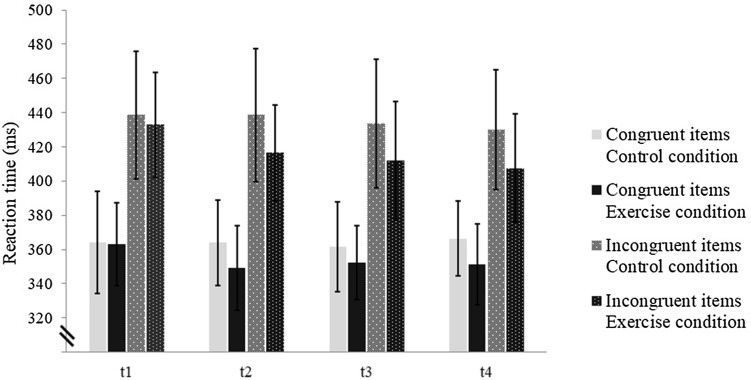
Reaction time of correct responses on congruent and incongruent stimuli for the control and exercise condition in the pre- (*t*1) and post blocks (*t*2–*t*4); reaction times on congruent and incongruent stimuli were significantly shorter after physical exercise, whereas no significant change was observed in the control condition

Regarding reaction time, a 3-way ANOVA (Condition [control, exercise] × Time [*t*1–*t*4] × Compatibility [congruent vs. incongruent]) yielded significant main effects of Condition, Time, and Compatibility (see Table 3). Reaction time was significantly longer in the control condition as compared to the exercise condition, and significantly shorter for congruent as for incongruent items, respectively. A significant interaction of Condition × Time was obtained, reflected by a strong decrease of reaction time from pre- (*t*1) to the first post-test (*t*2) and nearly no changes in the further post-tests (*t*3 and *t*4) in the exercise condition. However, in the control condition reaction time remained quite stable (see Table 3; Fig. 2). Subsequently, 2-way ANOVAs (Time [*t*1–*t*4] × Compatibility [congruent, incongruent]) were run for each condition separately indicating only a significant Time effect in the exercise condition, *F*(3, 33) = 8.79, *p* < .01, *η*_p_^2^ = 0.44. Post hoc analyses demonstrated significant shorter reaction times in the first post-exercise (*t*2; *p* = .02) and last post-exercise block (*t*4; *p* = .02), and a trend towards a significant difference in second post-exercise EF block (*t*3; *p* = .07) as compared with the pre-exercise block (*t*1). Furthermore, a Time × Compatibility effect was obtained in the exercise condition, *F*(3, 33) = 3.37, *p* < .05, *η*_p_^2^ = 0.23. While reaction time on congruent items decreased after exercise and maintained at a constant level in the subsequent EF blocks, reaction time on incongruent items decreased continuously from the first to the last block (see Table 3).

The number of errors on incongruent items yielded a significant Condition × Time effect (see Table 3). In the control condition, the number of errors decreased from the first to the third EF block (*t*3), and increased in the last EF block (*t*4). The number of errors in the exercise condition, however, demonstrated only small changes over time (see Table 3). Subsequently, separate ANOVAs for each condition demonstrated only a significant Time effect in the control condition, *F*(3, 33) = 3.85, *p* < .05, *η*_p_^2^ = 0.26. Post hoc analyses showed no significant differences between all measurements in the control condition (*p* > .16).

For the flanker effect, a significant result for Time was obtained (see Table 4). Post hoc tests revealed significantly reduced scores in the last post-test (*t*4) as compared with the pre- (*t*1; *p* = .03) and the first post-test (*t*2; *p* = .02).

**Table 4:** Results of the 2 × 4 × 2 ANOVA regarding reaction time on correct items and results of 2 × 4 ANOVAs regarding the number of errors on incongruent items and the flanker effect

	Condition				Time				Compatibility				Condition × time				Condition × compatibility				Time × compatibility			
	*df*	*F*	*η*_p_^2^	*p*	*df*	*F*	*η*_p_^2^	*p*	*df*	*F*	*η*_p_^2^	*p*	*df*	*F*	*η*_p_^2^	*p*	*df*	*F*	*η*_p_^2^	*p*	*df*	*F*	*η*_p_^2^	*p*
Reaction time on correct items	1, 11	13.70	0.56	< 0.01	3, 33	6.45	0.37	< 0.01	1, 11	140.99	0.93	< 0.001	3, 33	2.95	0.21	< 0.05	1, 11	3.94	0.26	0.07	3, 33	5.01	0.31	< 0.01
Errors on incongruent items	1, 11	1.06	0.09	0.33	3, 33	1.72	0.14	0.18					3, 33	3.21	0.23	< 0.05								
Flanker effect	1, 11	3.94	0.26	0.07	3, 33	5.01	0.31	< 0.01					3, 33	0.44	0.04	0.60								

### Electrocortical measures

Table 5 reports descriptive statistics of N2 and P3 components for congruent and incongruent stimuli separated for each condition.

**Table 5:** Descriptive statistics of N2 and P3 amplitudes, and latencies separated for each condition and compatibility

	Control condition				Exercise condition			
	Congruent		Incongruent		Congruent		Incongruent	
	*M*	SD	*M*	SD	*M*	SD	*M*	SD
N2 Amplitude (µV)								
*t*1	− 4.78	2.94	− 5.87	3.14	− 4.63	3.00	− 5.92	2.52
*t*2	− 5.01	1.73	− 5.50	2.89	− 3.08	3.00	− 4.47	3.24
*t*3	− 4.83	2.20	− 5.76	3.02	− 3.80	2.09	− 4.21	3.72
*t*4	− 3.80	2.01	− 4.59	2.34	− 4.10	2.56	− 3.81	2.48
N2 Latency (ms)								
*t*1	202.89	22.18	207.80	21.97	196.23	23.51	202.73	26.74
*t*2	201.35	21.82	200.29	24.57	191.14	24.02	195.75	23.36
*t*3	197.56	29.36	205.62	20.78	196.21	24.82	200.97	22.37
*t*4	205.00	25.03	198.83	16.57	194.09	29.06	201.77	22.73
P3 Amplitude (µV)								
*t*1	5.90	2.16	5.40	2.09	6.14	2.28	6.03	2.35
*t*2	5.72	2.21	6.01	1.90	6.28	2.09	5.83	2.27
*t*3	5.90	1.99	5.60	2.58	5.29	2.28	5.71	2.47
*t*4	5.42	2.25	5.07	2.22	5.81	1.86	6.60	2.00
P3 Latency (ms)								
*t*1	298.88	31.38	304.69	28.16	297.27	21.21	306.29	27.07
*t*2	291.79	26.96	304.33	32.76	263.58	20.88	296.73	39.48
*t*3	296.54	29.66	305.01	30.29	277.24	28.16	287.25	44.37
*t*4	283.50	25.09	300.90	31.76	275.32	22.20	294.95	21.20

Results of 2 × 4 × 2 ANOVAs for all ERP components are displayed in Table 6. The non-significant 2-way interactions of Time × Compatibility as well as non-significant 3-way interactions of Condition [control, exercise] × Time [*t*1–*t*4] × Compatibility [congruent vs. incongruent] are not included for reasons of clarity and comprehensibility.

**Table 6:** Main effects and interaction of condition × time of the 2 × 4 × 2 ANOVAs on amplitude and latency of N2 and P3

	Condition				Time				Compatibility				Condition x Time			
	*df*	*F*	*η*_p_^2^	*p*	*df*	*F*	*η*_p_^2^	*p*	*df*	*F*	*η*_p_^2^	*p*	*df*	*F*	*η*_p_^2^	*p*
N2 Amplitude	1, 11	10.20	0.48	< 0.01	3, 33	5.95	0.35	< 0.01	1, 11	5.77	0.34	0.04	3, 33	3.34	0.23	0.03
N2 Latency	1, 11	6.39	0.37	0.03	3, 33	1.18	0.10	0.33	1, 11	4.96	0.31	< 0.05	3, 33	0.23	0.02	0.88
P3 Amplitude	1, 11	0.37	0.03	0.56	3, 33	0.50	0.04	0.66	1, 11	0.01	0.001	0.93	3, 33	1.62	0.13	0.22
P3 Latency	1, 11	7.49	0.41	0.02	3, 33	3.90	0.26	0.02	1, 11	13.59	0.55	< 0.01	3, 33	3.40	0.24	0.03

The 2-way interactions of time × compatibility as well as 3-way interactions of condition × time × compatibility failed to reach significance

### N2 amplitude

**Fig. 3:**
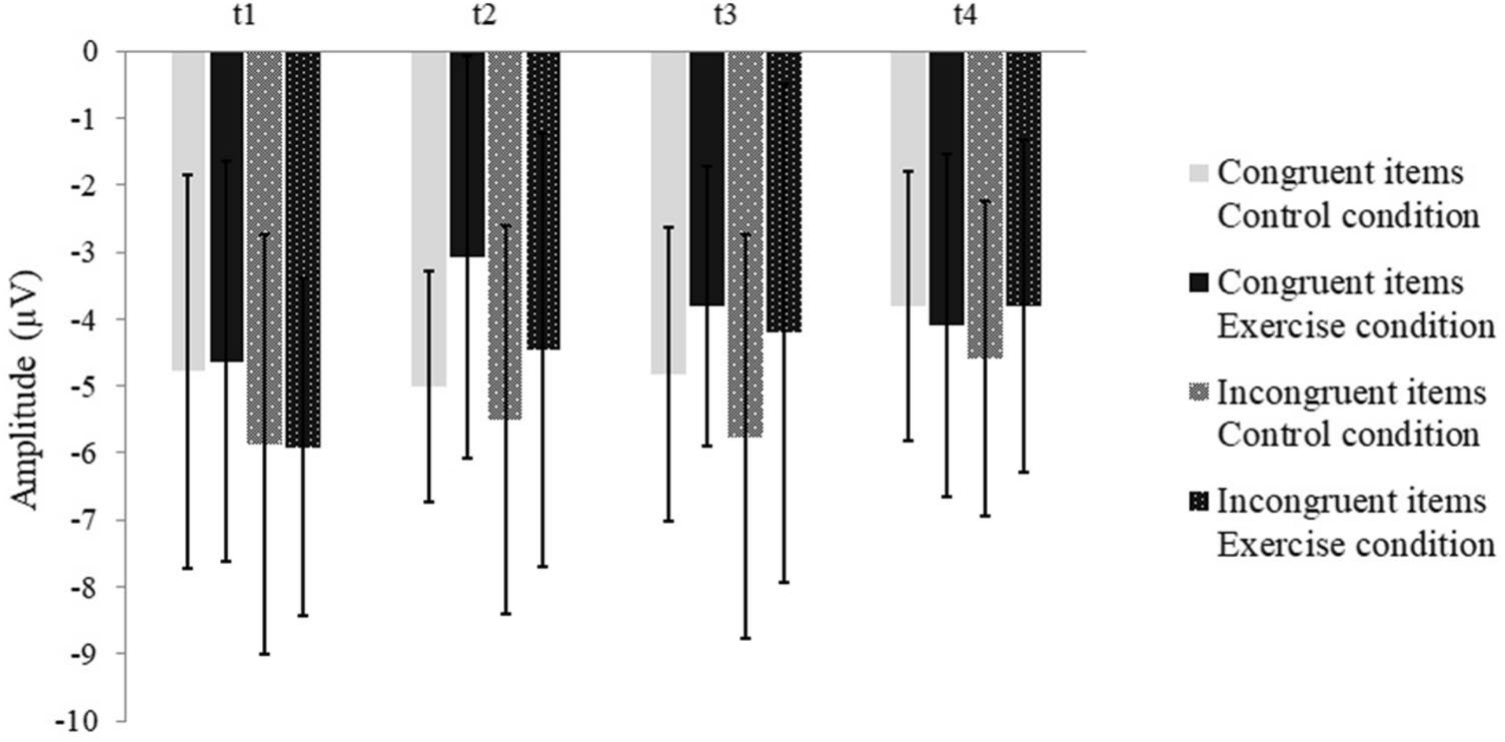
N2 amplitude of correct responses on congruent and incongruent stimuli for the control and exercise condition in the pre- (*t*1) and post- EF blocks (*t*2–*t*4); N2 amplitude on congruent and incongruent stimuli displayed a significant decrease following physical exhaustion, whereas N2 amplitude remained stable in the control condition

Analyses revealed a significant main effect of Condition, with larger N2 amplitudes in the control condition as compared to the exercise condition. Furthermore, a significant time effect was observed. Post hoc analyses indicated a significant decrease between the pre-test (*t*1) and the last post-test (*t*4; *p* = .03). A significant effect for Compatibility revealed smaller amplitudes for congruent than for incongruent items. In addition, a significant interaction of Condition × Time was found. In the exercise condition, N2 amplitude decreased from pre- (*t*1) to the first post-test (*t*2) and maintained the level to the last post-test (*t*4). In the control condition, the amplitude stayed stable in the first three EF blocks (*t*1–*t*3) and decreased at t4 to a similar level as in the exercise condition at t4 (see Table 5; Fig. 3). The subsequent ANOVAs separated for condition demonstrated a significant Time effect in the exercise condition, *F*(3, 33) = 6.31, *p* < .01, *η*_p_^2^ = 0.37, and no Time effect in the control condition. Post hoc analyses on differences regarding blocks in the exercise condition revealed a significant lower N2 amplitude in the first (*t*2; *p* = .03) and last post-exercise block (t4; *p* < .01) compared with pre-exercise (*t*1). No further significant interactions were observed.

### N2 latency

N2 latency was significantly longer in the control as in the exercise condition. Furthermore, a significant main effect of Compatibility was obtained with longer latencies for incongruent as compared to congruent items. No Time effect and no interaction effects were found.

### P3 amplitude

There were no significant main effects and no interactions in P3 amplitude.

### P3 latency

**Fig. 4:**
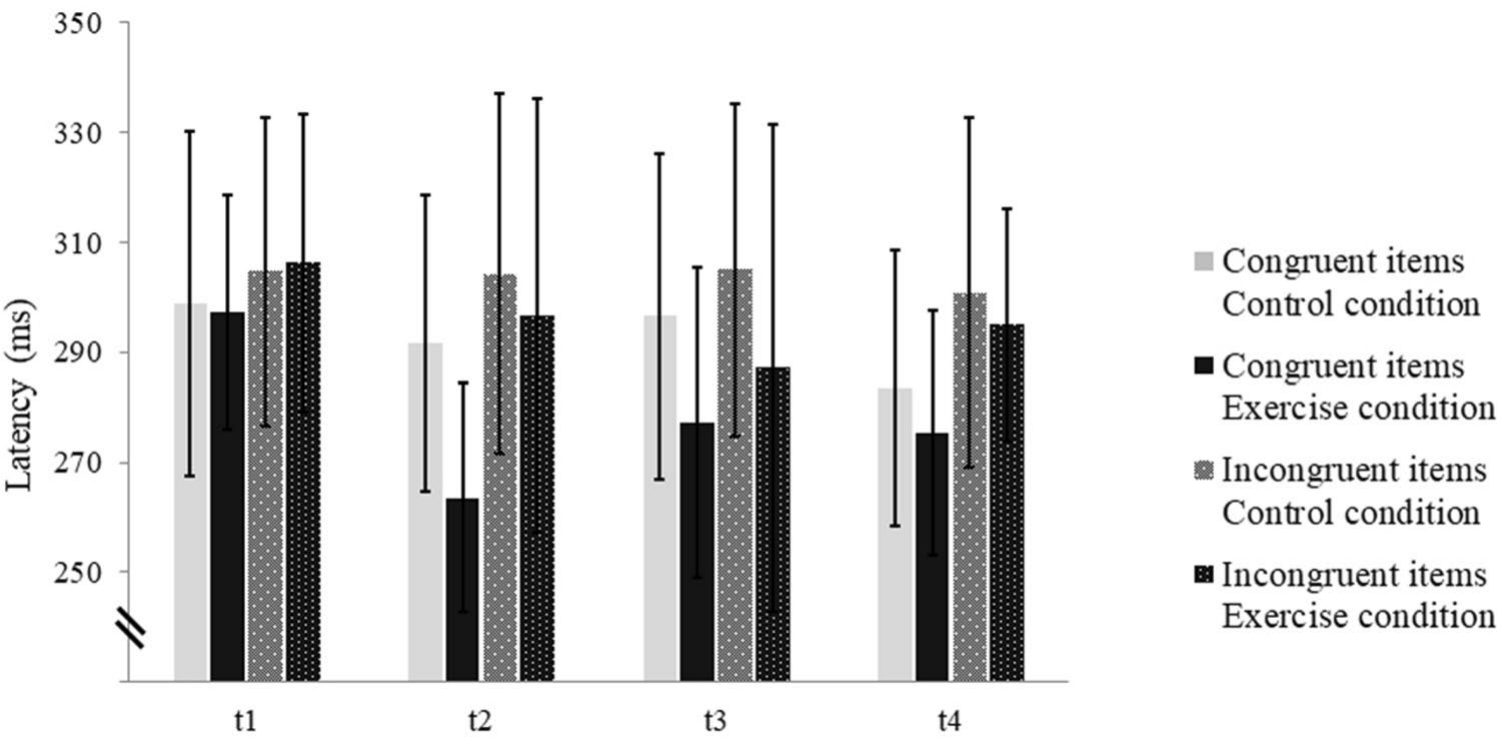
P3 latency of correct responses on congruent and incongruent stimuli for the control and exercise condition in the pre- (*t*1) and post- EF blocks (*t*2–*t*4); P3 latency demonstrated a significant Time effect in the exercise condition with shorter latencies following physical exercise; P3 latencies of the control condition maintained relatively stable

Analyses revealed a significant Condition effect showing longer latencies in the control condition as compared to the exercise condition. Moreover, a significant Time effect was observed. Subsequent post hoc tests failed to reach significance. Additionally, a significant Compatibility effect occurred, displaying significant shorter latencies on congruent than on incongruent items. Finally, an interaction effect of Condition × Time was obtained. In the exercise condition, P3 latency declined by 20 ms from pre- exercise (t1) to the first post-exercise block (*t*2) and demonstrated marginal changes in the following blocks (*t*3 and *t*4). P3 latencies of the control condition maintained relatively stable across the first three EF blocks (*t*1–*t*3) and decreased slightly at t4 (see Table 5; Fig. 4). The subsequent ANOVA for the exercise condition revealed a significant Time effect, *F*(3, 33) = 4.01, *p* = .05, *η*_p_^2^ = 0.27. Post hoc analyses marginally failed to reach significance between the pre-exercise block (*t*1) and the first (*t*2; *p* = .06) as well as the second post-exercise block (*t*3; *p* = .06). ANOVA on the control condition demonstrated no significant Time effect. No more significant interaction effects were observed.

## Discussion

In competitive sports, a wealth of information has to be gathered and processed in recovery phases. This study aimed at examining the impact of a maximal physical exertion on interference control with respect to changes of behavioural and electrocortical measures in the time course of recovery. Previous research has paid some attention to cognitive processes, in particular to cognitive control, immediately following maximal physical exhaustion as well as after short and medium recovery time. Recent research suggested that a state of maximal physical exhaustion is associated with an increased reaction speed and impaired divided attention (Coco et al. 2009), impaired exogenous spatial attention (Llorens et al. 2015), accelerated speed discrimination at cost of accuracy (Thomson et al. 2009), and deteriorated basic neuropsychological functions (Covassin et al. 2007). However, research neglected to evaluate the impact of the length of recovery on cognitive processes. The current findings revealed that acute bouts of maximal physical load decreased information processing time in the post-exercise EF blocks (*t*2–*t*4) regardless of compatibility mode (congruent, incongruent). Physical exhaustion had no impact on accuracy, and on the flanker effect. N2 amplitude was reduced, and P3 latency turned out to be shorter compared to a control condition in both compatibility modes in the time course of the recovery phase (*t*2–*t*4). Regarding differences between post-exercise EF blocks, no empirical evidence was found for a different impact of the recovery duration on interference control, and brain activity.

The moderate levels of HR during the post-exercise EF tasks ranging between 125 and 98 beats/min (see Table 3) are in line with arousal theories, predicting that cognitive performance rises to an optimal level as exercise-induced arousal increases and declines when higher levels of physiological arousal are reached (Lambourne and Tomporowski 2010). The high fitness level of the participants may account for the fast recovery of the physical stressor to a state of moderate arousal, which might be associated with increases in information processing speed. The observed changes in reaction time are in discrepancy to results of Kamijo et al. (2004b) who found no changes in pre-motor time in a go/no-go task following maximal physical exhaustion compared to a control condition. Kamijo et al. (2004b), however, administered a go/no-go task that requires to react on a target signal or to withhold a reaction, and thus appears to be less demanding on executive functions than a flanker task. Thus, maximal physical exhaustion might influence sub-domains of inhibitory control in different ways.

Regarding changes during the recovery phase, weak, but not significant, alterations in reaction time were observed. The difference of physiological arousal among the three post-exercise measurements could be too low to affect reaction time differently. The most pronounced, but not significant changes in reaction time on incongruent items could indicate that the cognitively more demanding task is more sensitive to alterations in the physiological state in the recovery phase following maximal physical exhaustion. This is in line with the assumptions that tasks requiring executive functions are predominantly influenced by exercise (Chodzko-Zajko 1991). Partial support for this assumption is given by O’Leary et al. (2011) who found a significant decrease of reaction time interference (RT incongruent-RT congruent) 20 min following treadmill walking at 60% HR_max_ for 20 min relative to seated rest and videogame play. Future studies, however, should investigate changes of interference control as indexed by the flanker score. When analysing the flanker score, it is proposed to consider whether alterations are a result of reaction time changes on congruent or incongruent items or even by a change of both.

In accordance with expectations, results yielded no impact of maximal physical load error rate, suggesting that no speed-accuracy trade-off may have favoured improvements in information processing speed. This finding is in line with those of Kamijo et al. (2007) who, however, used a go/no-go task. It is most likely that go/no-go and flanker tasks are too simple to elicit a cognitive decline as proposed by Ando et al. (2011). Consequently, the implementation of more complex executive function tasks is recommended when interested in blunders under the impact of high cardiovascular load.

Regarding the N2 component, the current findings are well in line with previous studies showing larger N2 amplitudes on incongruent trials than congruent trials (Kopp et al. 1996; Heil et al. 2000; Pontifex and Hillman 2007), as well as longer N2 latencies on incongruent trials than congruent trials (Yeung et al. 2004; Pontifex and Hillman 2007). The novelty of this study is the recording of N2 components in a flanker task following maximal physical load, which represents a new approach to extend knowledge of the underlying processes related to the resolution of a reaction conflict in a state of high physiological arousal. To date, only two studies examined the impact of exercise on cognitive control using the flanker task. In contrast to this study, however, the cognitive task was administered during exercise (Pontifex and Hillman 2007; Olson et al. 2016) leading to discrepant findings of the effects of exercise on N2 amplitude in a flanker task, which is discussed as a result of using different research designs (Olson et al. 2016). Hence, it is difficult to draw conclusions of those studies regarding the actual findings. The smaller N2 amplitude during the post-exercise EF blocks of the current study may reflect a reduced conflict monitoring, suggesting a detrimental effect of maximal physical exercise on cognitive control. Although the observed reduced N2 amplitude is not associated with an impaired behavioural performance in the number of errors on incongruent stimuli, the significant interaction of time and condition could be considered as a sign of a possible effect of maximal physical load on response accuracy in incongruent items. In the control condition, errors on incongruent items yielded a significant change across the four measurements, characterized through a decrease of errors in the second and third block in comparison to the first EF block (see Table 3). However, it has to be pointed out that post hoc analyses revealed no significant difference among measurements.

The cognitive processing speed of reaction inhibition as indexed by N2 latency (Pontifex and Hillman 2007) appears to be unaffected by a preceding maximal physical load. Looking at the descriptive statistics, the shorter mean of N2 latency after incongruent stimuli compared to congruent stimuli at t2 and t4 in the control condition has to be discussed. It is suggested that the reduced N2 latency at these two measurements could reflect a change in the strategy of processing the flanking arrows. Possibly, the recovery period prior to the second and the fourth EF block could stimulate adaptation processes. The strategy could be to improve performance on incongruent stimuli, which may lead to a faster detection of the response conflict on incongruent stimuli. Small support for this assumption regarding the control condition comes from a reduced reaction time on incongruent stimuli, and a nearly unchanged reaction time on congruent stimuli at t4 in comparison to t1. An explanation that this effect was not observed in the exercise condition could be that the physical load pools resources for coping with the physical stressor. Consequently, adaptations to the task through task repetition are prevented.

The current findings on P3 latency replicate earlier studies with an increased P3 latency in incongruent trials compared to congruent trials (Pontifex and Hillman 2007). Regarding P3 amplitude, Pontifex and Hillman (2007) found a larger amplitude for congruent items than for incongruent, whereas Olson et al. (2016) observed reversed findings. This study demonstrated no difference between congruent and incongruent items in P3 amplitude. Different sample characteristics might have resulted in deviant findings. Thus, it is recommended to clarify determining factors on the relationship of P3 amplitude and compatibility mode in future studies. During the post-exercise phase, P3 latencies were significantly shorter in congruent and incongruent trials reflecting a general effect on stimulus classification speed, which goes along with faster reaction times. This is in contrast to Kamijo et al. (2007) who found that P3 latency of incongruent trials is more sensitive to a preceding light, moderate or submaximal physical load than neutral trials requiring less executive control. Different physical loads and different compatibility modes of the flanker tasks may explain the discrepant findings. Consequently, further studies should address the effect of exercise on P3 latency in tasks requiring varying levels of inhibitory control.

Immediately after physical load (*t*2) as well as during recovery (*t*3 and *t*4), P3 amplitude yielded no change in comparison to baseline, suggesting that the amount of attentional resources devoted to the flanker task may not be influenced by maximal physical load and by a recovery phase of 15 min. Findings on P3 amplitude immediately after maximal physical exhaustion appear to be in line with the inverted U shaped function of exercise intensity and P3 amplitude as proposed by Kamijo et al. (2007). According to that model, however, P3 amplitude was expected to increase as consequence of a reduced physiological arousal during the recovery phase, and not to remain stable. One possible explanation for this result could be found in the need of more recovery time to reduce arousal to a level at which an increase of P3 amplitude will occur. Thus, more research should focus on changes of P3 amplitude after exercise during short, medium and long-term recovery periods.

Finally, research can benefit from the application of more sophisticated cognitive control tasks (e.g. complex flanker task consisting of more target stimuli and a lower occurrence rate of incongruent stimuli) provoking more errors to prove whether changes in neuroelectric measures are associated with behavioural ones. So far, most of the research on exercise and cognition failed to deliver evidence for this relationship. To sum up, results of this study provide evidence that sportspersons do not only tolerate, but even profit from a preceding maximal physical exertion in terms of information processing in a cognitive control task that remains during a recovery period of 15 min. Thus, cognitive control in biathlon shooting could be positively influenced by previous physical load in biathlon. Control processes that are necessary to follow coaches’ instructions at halftime may benefit from prior intense exercise.

### Limitations

First, findings of this study are limited to interference control and selective attention as measured by the flanker task. However, there is weak evidence of a specific impact on executive functions through preceding maximal physical load (Kamijo et al. 2007). Thus, it is recommended to examine other sub-domains of executive functioning in future studies. Second, a problem for interpreting the effects on the analysed N2/P3 complex is that although the amplitude and latency of a latent component are conceptually independent, they are often confounded (for a more detailed discussion on this issue see Luck 2005). Thus, exercise-induced changes in P3 latency might have resulted from changes of the N2 component. Despite this alternative explanation, however, the assumption of an improved stimulus classification speed is strongly supported by the behavioural findings on reaction time. Third, the selected sample of well-trained persons showed a large variation in the time until they reached a state of voluntary physical exhaustion. However, there was no evidence that different fitness levels within this sample had an effect on the results. Nevertheless, the findings cannot be transferred to untrained persons or elite athletes. It could be that persons of lowest or highest fitness levels cope differently with a preceding maximal physical stressor. Fourth, the repetitive administration of the EF task could have elicited cognitive fatigue. However, the study design, the short duration of the flanker task, and the recovery phases each between the first and the second, as well as the third and the fourth EF block argue against the possibility that cognitive fatigue has influenced the findings.

## Conclusions

This study extends knowledge of the small body of literature that has examined cognitive performance immediately after maximal physical exhaustion and during recovery. Well-trained persons appear not only to withstand, but even to benefit from preceding maximal physical exhaustion with respect to inhibitory processes and selective attention during a recovery phase of 15 min. Analyses of ERP components suggest that the observed improvements in information processing speed go along with a reduced conflict monitoring and a decreased stimulus classification speed. In general, the analyses of executive functions in a state of maximal physical exertion have the potential to contribute to a deeper understanding of the interplay of exercise and cognition. From an applied perspective, the evaluation of executive functions in extreme situations as in a state of maximal physical exertion may contribute to improvements of sport psychological diagnostics and interventions.

## Abbreviations

ANOVA: Analysis of variance
BL: Baseline
ECG: Electrocardiogram
EEG: Electroencephalogram
EF: Eriksen flanker
EOG: Electrooculogram
ERP: Event-related potential
HR: Heart rate
HR_max_: Maximal heart rate
RPE: Rate of perceived exertion
*V*O_2max_: Maximal oxygen uptake
*W*_max_: Maximal power output
